# A multivariate approach to investigate the combined biological effects of multiple exposures

**DOI:** 10.1136/jech-2017-210061

**Published:** 2018-03-21

**Authors:** Pooja Jain, Paolo Vineis, Benoît Liquet, Jelle Vlaanderen, Barbara Bodinier, Karin van Veldhoven, Manolis Kogevinas, Toby J Athersuch, Laia Font-Ribera, Cristina M Villanueva, Roel Vermeulen, Marc Chadeau-Hyam

**Affiliations:** 1 Department of Epidemiology and Biostatistics, School of Public Health, MRC-PHE Centre for Environment and Health, Imperial College London, London, UK; 2 Molecular and Genetic Epidemiology Unit, Italian Institute for Genomic Medicine (IIGM), Turin, Italy; 3 UMR CNRS 5142, Laboratoire de Mathématiques et de leurs Applications, Université de Pau et des Pays de l’Adour, Anglet, France; 4 School of Mathematics, ARC Centre of Excellence for Mathematical and Statistical Frontiers, Queensland University of Technology, Brisbane, Australia; 5 Institute for Risk Assessment Sciences, Division of Environmental Epidemiology, Utrecht University, Utrecht, Netherlands; 6 ISGlobal, Centre for Research in Environmental Epidemiology (CREAL), Barcelona, Spain; 7 Universitat Pompeu Fabra (UPF), Barcelona, Spain; 8 CIBER Epidemiología y Salud Pública (CIBERESP), Barcelona, Spain; 9 IMIM (Hospital del Mar Medical Research Institute), Barcelona, Spain; 10 Division of Computational and Systems Medicine, Department of Surgery and Cancer, Faculty of Medicine, Imperial College London, London, UK

**Keywords:** exposome, multiple exposures, multivariate response, OMICs data, multi-level sparse PLS models

## Abstract

Epidemiological studies provide evidence that environmental exposures may affect health through complex mixtures. Formal investigation of the effect of exposure mixtures is usually achieved by modelling interactions, which relies on strong assumptions relating to the identity and the number of the exposures involved in such interactions, and on the order and parametric form of these interactions. These hypotheses become difficult to formulate and justify in an exposome context, where influential exposures are numerous and heterogeneous. To capture both the complexity of the exposome and its possibly pleiotropic effects, models handling multivariate predictors and responses, such as partial least squares (PLS) algorithms, can prove useful. As an illustrative example, we applied PLS models to data from a study investigating the inflammatory response (blood concentration of 13 immune markers) to the exposure to four disinfection by-products (one brominated and three chlorinated compounds), while swimming in a pool. To accommodate the multiple observations per participant (n=60; before and after the swim), we adopted a multilevel extension of PLS algorithms, including sparse PLS models shrinking loadings coefficients of unimportant predictors (exposures) and/or responses (protein levels). Despite the strong correlation among co-occurring exposures, our approach identified a subset of exposures (n=3/4) affecting the exhaled levels of 8 (out of 13) immune markers. PLS algorithms can easily scale to high-dimensional exposures and responses, and prove useful for exposome research to identify sparse sets of exposures jointly affecting a set of (selected) biological markers. Our descriptive work may guide these extensions for higher dimensional data.

## Introduction

Health effects of simultaneous exposure to numerous and possibly interacting chemicals is raising growing public health concerns,^1–3^ and such investigation calls for the definition of efficient statistical approaches.^4^ The exploration of the biological responses to external exposures, as formalised in the exposome concept,^5–9^ relies on statistical methods accommodating the multivariate and complex interrelations of exposures. Most of the proposed supervised methods rely on dimensionality reduction or (Bayesian) variable selection.^10 11^ These methods can handle the multidimensionality of exposome data as well as existing correlation structures. However, in practice, the use of these methods has been so far mainly restricted to the exploration of a single exposure and/or endpoint at a time. Supported by the established and possibly differential combination of exposures to which populations are subjected,^12 13^ developing statistical approaches to investigate the effect of mixtures has represented a further active research field and resulted in methods explicitly modelling interactions between exposures.^14–16^ Their usability, however, remains limited in an agnostic context, due to dimensionality and interpretability issues.^4 17^ Furthermore, these approaches rely on strong assumptions mainly relating to the number, the order, and parametric form of the interactions between exposures. These assumptions become even more complex to formulate and to justify when, as generally the case in exposome studies, most of the effective exposures and their effects are not measurable and even sometimes unknown/unidentified.

To address the dimensionality burden of including interaction terms in the statistical models, a two-stage strategy first identifying prioritised exposures and second investigating their potential interactions has been proposed.^17 18^ However, this approach assumes that the most relevant exposures potentially active in a mixture could be detected based on their marginal effects or correlation. In order to better capture the complexity of the exposure mix, including potential (un-modelled) interactions, models accommodating multivariate exposures are needed. Furthermore, to account for complex and possible pleiotropic effects of these exposures, multivariate responses need to be accounted for. As a supervised dimensionality reduction technique, partial least squares (PLS) regression aims at constructing summary latent variables as linear combinations of the original predictors and response variables. These summary variables are constructed not only so that they capture as much information as possible in each block of data, but also identifies (i) the variability in the predictors that is relevant to the outcomes, and (ii) the variability in the responses which is mostly linked to the predictors.^19 20^ Variable selection can also be achieved in PLS regression through L1 penalisation shrinking towards 0 the loadings coefficients of the least influential/influenced variables.^21–23^

In order to accommodate repeated measurements, linear mixed models or multivariate normal models^24 25^ can be used in an univariate context, and for dimensionality reduction techniques, multilevel approaches have been proposed.^26^

As a description of the application of multilevel PLS regression approaches and their sparse variants to investigate the multivariate response to multivariate exposures in the presence of multiple measurements per participants, we analyse here the inflammatory response to the exposure to a set of four trihalomethanes (THMs): chloroform (CHCl_3_), bromodichloromethane (BDCM), dibromochloromethane (DBCM), bromoform (CHBr_3_), measured in exhaled breath as a surrogate for the exposure to disinfection by-products (DBP). The panel of immune markers we assay include interleukins (IL), growth factors, (C-C and C-X-C motif) chemokines ligands, IL receptor antagonists and C reactive proteins (CRP), which may be involved in the reported immunotoxic effect of DBPs and similar compounds^27 28^ and/or in the anti-inflammatory effects of physical exercise.^29^

## Methods

Data were collected within the ‘EXPOsOMICS’ project^30^ and include measurements of (i) four DBPs in exhaled breath from participants before and after a swimming session in a chlorinated pool (PISCINA II study) and (ii) at both time points, blood levels of (n=13) inflammation-related proteins.^28^

### Study population

The PISCINA II study is an experimental investigation of the acute biological response to exposures induced by swimming in a chlorinated pool. As detailed elsewhere,^31^ the study included volunteers, aged 18–40 years, non-smoking and non-professional swimmers, who swam for 40 min in a 25 m long indoor chlorinated pool in Barcelona, Spain, between June and December 2013. At the time of the experiment, participants were asked to complete a questionnaire providing information on sociodemographic, dietary habits, regular physical activity, medical and anthropometric factors.

DBPs including four THMs, CHCl_3_, BDCM, DBCM and CHBr_3_, were measured in exhaled breath at two time points: before swimmers entered the swimming pool and immediately after they exited the swimming pool, using the Bio-VOC Sampler (Markes International Ltd, UK). These chemicals were assessed by gas chromatography coupled to a mass spectrometer. Details on sampling collection and analysis have been published previously.^31^

For each of these 60 participants with full exposure and questionnaire data, two blood samples collected before and 2 hours after swimming were available. These were collected in a room detached from the swimming pool area and stored at −80°C.

Informed consent was provided by each participant before commencement of the experiment.

### Protein assay

As detailed elsewhere,^28^ a panel of 23 immune markers from both serum samples was assessed using an R & D Systems (Abingdon, UK) Luminex screening assay according to the protocol described by the manufacturer. The panel includes interleukin (IL)-1β, IL-1rA, IL-4, IL-5, IL-6, IL-8, IL-10, IL-13, IL-17, tumour necrosis factor-alpha (TNF-α), epidermal growth factor (EGF), macrophage inflammatory protein 1 beta (MIP1 β), chemokine (C-X-C motif) ligand 1 (CXCL1), myeloperoxidase (MPO), C-X-C motif chemokine 10 (CXCL10), vascular endothelial growth factor (VEGF), C-C motif chemokine 22 (CCL22), periostin, chemokine (C-C motif) ligand 2 (CCL2), basic fibroblast growth factor (FGF basic), granulocyte colony-stimulating factor (G-CSF) and C-C motif chemokine 11 (CCL11). In addition, CRP was assessed using an R & D Systems Solid Phase Sandwich ELISA. Both samples from the same individual were analysed in the same analytical batch. Serum concentrations for IL-1β, IL-4, IL-5, IL-6, IL-10, IL- 13, TNF-α, MIP1 beta, CXCL1, and FGF basic were below the limits of quantification in more 60% of the samples and were therefore excluded from the analyses. Missing values in the remaining 13 immune markers were imputed using a maximum likelihood estimation procedure.^32^

### Statistical model

Descriptive analyses of the data were done using paired Student’s t-test comparing the mean concentration of exposures (log-transformed) and proteins before and after the swimming experiment.

We used a partial least squares (PLS) approach in regression mode, setting the (n=4) exposures as predictors and the (n=13) proteins as multivariate response variables. PLS algorithms determine components as linear combinations of predictors X (exposures) and responses Y (proteins) maximising their variance-covariance. Denoting T and U the resulting projections of X and Y, respectively, and P and Q the loadings matrices, the PLS regression decomposes X and Y as

$$\left\{ \begin{array}{l} X=TP^{T}+E\\ Y=UQ^{T}+F \end{array}\right.$$

Where E and F are error terms assumed to be independent and identically distributed Gaussian variables. In this supervised context, latent variables of X capture the variability in exposures that is relevant to the inflammatory profile, and symmetrically, the components of Y capture the variability in the inflammatory profiles that is related to exposures.

The contribution of each of the original variables in defining the PLS components (loadings coefficients) enables to identify which (combination of) exposures drive the X-Y variance covariance structure and can be used to identify the (sets of) exposures mostly related to the (sets of) inflammatory proteins. The proportion of variance in X (or Y) explained by a given component of X (or Y) measures how accurately that single component summarises the information contained in the original X (or Y) matrix. The percentage of variance of Y explained by the components of X measures relevance of information summary provided by the PLS components of X to the outcome matrix.

Variable importance in projection (VIP) measures, for a given X component, the relative contribution of each of the original predictor to the explanation of the X-related variance in the outcome. VIP thereby measures the contribution of each original predictors in the overall explanatory performance of a given X component, and helps identifying the main drivers of that component.^33^ Typically, a variable with a VIP >1 is considered explanatory for the variation in Y captured by that component.

To accommodate the repeated measure design, before and after swimming, where both exposure and protein levels are available, we adopted a multilevel approach, which decomposes the observed variability into within-individual and between-individual variability. The within-individual variability (ie, changes induced, within each participant, by the experiment) is included into a standard PLS model to identify linear combinations of exposures that are best able to explain within individual changes in inflammatory markers levels.

We ran our multilevel PLS analyses setting the four exposures predictors, and the 13 proteins as outcome. Four variants of the PLS approach were considered: the natural PLS, and sparse PLS (sPLS) imposing sparsity in the loadings coefficients of (i) X (exposures) components (sPLS_X_), (ii) Y (exposures) components (sPLS_Y_), (iii) both components of X and Y (sPLS_XY_). sPLS_X_ models shrink the X loadings coefficients towards 0 for the least informative exposure and hence help identifying the most relevant exposures with respect to inflammatory profiles. Symmetrically, variable selection of Y (sPLS_Y_) selects the proteins whose expression is mostly affected by exposures.

Calibration of the sPLS models was done using fivefold cross-validation which was independently repeated 1000 times. The cross-validation procedure is repeated for possible values of (i) the number of components to select and, (ii) the number of non-zero loadings coefficients (ie, the number of original variables contributing to the component). The number of components to be considered was determined using the average Q^2^ statistic calculated across all folds and repeats and was defined as the maximal the number of components such that adding an additional component would yield a substantive drop in the Q^2^ value. Sparsity of the sPLS models was controlled by setting the number of variable included to the one minimising the cross-validated prediction error.

Analyses were performed using R V.3.4.0 (21 April 2017). R-codes used for the analyses are available on request to the corresponding author.

## Results

### Exposure and protein data

Higher plasma proteins levels after swimming were only observed for Periostin and IL-1ra (table 1). Of the 13 assayed proteins, only 3 showed nominally significant difference (p<0.05) after the swimming experiment (CCL11, and CXCL10 lower and IL-1ra, higher).

**Table 1:** Summary statistics of PISCINA II study—mean (SD) levels of exposures and proteins before and after swimming

	Before swimming	After swimming	P values
	n=56	n=56	
Exposures in exhaled breath (μg/m^3^)			
CHCl_3_	0.43 (0.30)	11.53 (4.83)	7.0E−20
BDCM	0.06 (0.05)	2.49 (1.23)	7.0E−20
DBCM	0.02 (0.03)	0.54 (0.33)	1.3E−19
CHBr_3_	0.03 (0.02)	0.11 (0.08)	3.6E−16
Outcome (proteins concentration in pg/mL)			
CCL11	131.30 (30.86)	121.72 (29.36)	0.038
CCL2	214.92 (86.21)	204.52 (89.67)	0.420
CCL22	473.54 (195.94)	442.82 (193.87)	0.330
CRP	1616 (3,084.76)	1559.74 (3,081.06)	0.771
CXCL10	22.75 (11.44)	19.79 (10.27)	0.049
EGF	33.76 (26.20)	33.19 (34.37)	0.213
G-CSF	12.71 (6.44)	12.53 (6.66)	0.864
IL-17	4.79 (2.10)	4.74 (1.86)	0.877
IL-1ra	351.05 (193.27)	424.81 (252.39)	0.030
IL-8	4.44 (2.77)	3.77 (2.04)	0.265
MPO	12 885.16 (8,998.80)	12 829.78 (6,520.99)	0.248
Periostin	127,536.87 (39,857.79)	129,579.87 (41,388.81)	0.830
VEGF	54.76 (44.71)	52.48 (42.03)	0.841

* Differences in the mean levels before and after the swimming experiment are assessed using a paired Student t -test (log-transformed exposures values were considered).

BDCM, bromodichloromethane; CCL11, C-C motif chemokine 11, CCL2 motif, chemokine (C-C motif) ligand 2; CCL22, C-C motif chemokine 22; CHBr_3_, bromoform; CRP, C reactive protein;, CHCl_3_, chloroform; CXCL10, C-X-C motif chemokine 10, DBCM, dibromochloromethane; EGF, epidermal growth factor, G-CSF, granulocyte colony-stimulating factor, IL, interleukin; MPO, myeloperoxidase; VEGF, vascular endothelial growth factor.

**Figure 1:**
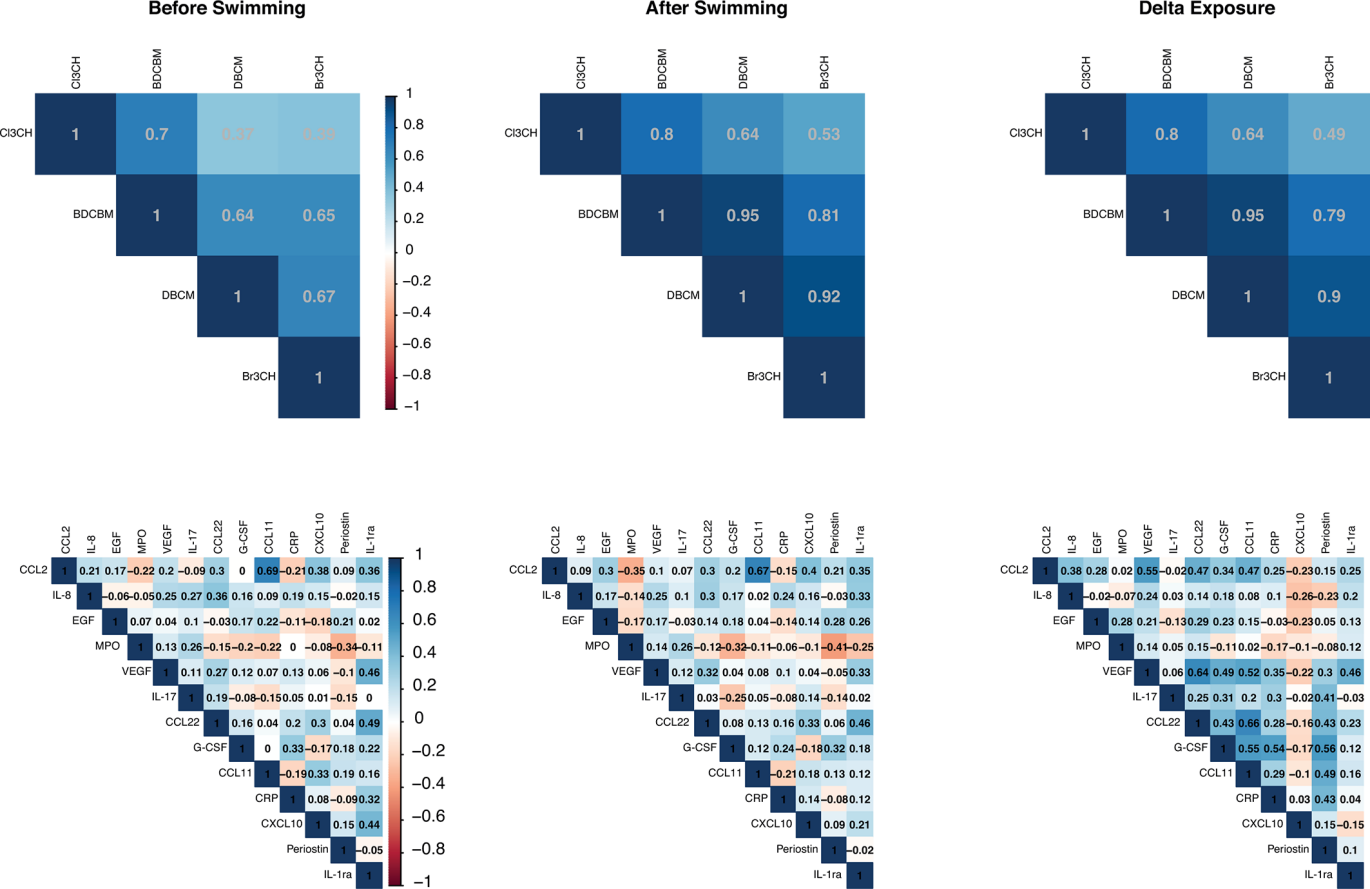
Spearman correlation coefficients for exposures (top) and Pearson correlation coefficients for protein levels (bottom) before (first column) and after (second column) the swim. The third column represent the correlation coefficients between differences in exposures and proteins levels. BDCM, bromodichloromethane; CCL11, C-C motif chemokine 11, CCL2 motif, chemokine (C-C motif) ligand 2; CCL22, C-C motif chemokine 22; CHBr_3_, bromoform; CRP, C reactive protein; CHCl_3_, chloroform; CXCL10, C-X-C motif chemokine 10; DBCM, dibromochloromethane; EGF, epidermal growth factor, G-CSF, granulocyte colony-stimulating factor; IL, interleukin; MPO, myeloperoxidase; VEGF, vascular endothelial growth factor.

For all four exposures, a strong contrast was observed before and after the swimming session (p values <4×10^−16^). Strong correlations among exposures were also observed, particularly in the postswimming samples (figure 1). Protein levels showed moderate correlation levels that were consistent before or after the swimming session (figure 1).

## PLS analyses

By construction, PLS models can calculate up to min(p_X_,p_Y_) components, where p_X_ and p_Y_ are the number of variables in the X and Y matrices, respectively. The loadings coefficients of the resulting 4 PLS components are reported in table 2, where (C_1X_,…,C_kX_) are the sets of k components for the predictor (exposure) matrix, and C_1Y _,…,C_kY _, the set of components relating to the response (proteins) matrix.

**Table 2:** Results from the multilevel (s)PLS analyses regressing the four exposures (predictors) against the 13 assayed proteins (response). Results are presented for the PLS analyses, for sparse PLS models performing variable selection on exposures (sPLS on X), on proteins (sPLS on Y) and on both exposures and proteins sPLS on X and Y. We report in the table the loadings coefficients for the (s)PLS components of exposures (top table) and proteins (bottom table). For the (sPLS) components of exposures, we report the per-component proportion of variance (in both X and Y) explained, and for components of the proteins we only report the proportion of the variance in Y. For all sparse PLS models, results are only presented for the first PLS component, which is the only one to be retained according to the Q^2^ criterion (see methods)

	PLS				sPLS on X	sPLS on Y	sPLS on X and Y
Exposures (X matrix)	C _1X_	C _2X_	C _3X_	C _4X_	C _1X_′	C _1X_″	C _1X_‴
CHCl_3_	− 0.50	− 0.60	− 0.60	− 0.17	− 0.48	− 0.50	-0.48
BDCM	−0.52	−0.21	0.45	0.70	−0.67	−0.52	−0.66
DBCM	−0.51	0.11	0.51	−0.68	−0.57	−0.51	−0.58
CHBr_3_	−0.46	0.76	−0.42	0.15	0.00	−0.46	0.00
Explained Variance in X	94.8%	4.5%	0.6%	0.04%	94.0%	94.8%	94.0%
Explained Variance in Y	10.1%	1.3%	1.9%	1.3%	10.4%	14.2%	16.1%
Protein levels (Y matrix)	C_1Y_	C_2Y_	C_3Y_	C_4Y_	C_1Y_'	C_1Y_'’	C_1Y_'’’
CCL2	0.12	0.195	−0.09	−0.02	0.13	0.00	0.00
IL-8	0.31	0.062	0.19	0.12	0.32	0.30	0.29
EGF	−0.10	0.216	−0.38	−0.11	−0.09	0.00	0.00
MPO	−0.14	0.310	0.18	0.05	−0.13	−0.02	0.00
VEGF	0.21	−0.266	−0.11	−0.36	0.20	0.13	0.11
IL-17	0.03	0.169	0.20	0.22	0.03	0.00	0.00
CCL22	0.42	−0.131	−0.32	−0.09	0.41	0.44	0.43
G-CSF	0.05	−0.079	−0.41	−0.43	0.05	0.00	0.00
CCL11	0.29	0.221	−0.27	−0.16	0.30	0.26	0.26
CRP	0.19	0.367	−0.11	−0.53	0.20	0.09	0.11
CXCL10	0.57	0.121	−0.05	0.46	0.57	0.68	0.67
Periostin	−0.18	−0.318	−0.31	−0.08	−0.18	−0.08	−0.08
IL-1ra	−0.38	−0.627	0.52	−0.28	−0.40	−0.39	−0.41
Explained Variance in Y	19.7%	6.9%	19.5%	23.3%	19.8%	17.7%	17.4%

Results are presented for the PLS analyses, for sparse PLS models performing variable selection on exposures (sPLS on X), on proteins (sPLS on Y) and on both exposures and proteins sPLS on X and Y. We report in the table the loadings coefficients for the (s)PLS components of exposures (top table) and proteins (bottom table). For the (sPLS) components of exposures, we report the per-component proportion of variance (in both X and Y) explained, and for components of the proteins we only report the proportion of the variance in Y. For all sparse PLS models, results are only presented for the first PLS component, which is the only one to be retained according to the Q ^2^ criterion (see methods).

BDCM, bromodichloromethane; CCL11, C-C motif chemokine 11, CCL2 motif, chemokine (C-C motif) ligand 2; CCL22, C-C motif chemokine 22; CHBr_3_, bromoform; CRP, C reactive protein; CHCl_3_, chloroform; CXCL10, C-X-C motif chemokine 10; DBCM, dibromochloromethane; EGF, epidermal growth factor, G-CSF, granulocyte colony-stimulating factor; IL, interleukin; MPO, myeloperoxidase; PLS, partial least squares; sPLS, sparse partial least squares; VEGF, vascular endothelial growth factor.

### PLS models

Owing to the strong correlation among exposures, a single component (C_1X_) for the exposure matrix is sufficient to explain about 95% of the variation in X. Each exposure contributed consistently to C_1X_: the four loadings coefficients were similar, though slightly lower (in absolute value) for bromoform. C_1X_ explained around 11% of the variance in Y, suggesting that most of the variation in exposure is able to capture a limited fraction of the variation in protein levels. The remaining 3 components of X explained between 4.33% and 0.04% of the variance in X, and these explained 1.33% to 1.87% of the variance in Y.

**Figure 2:**
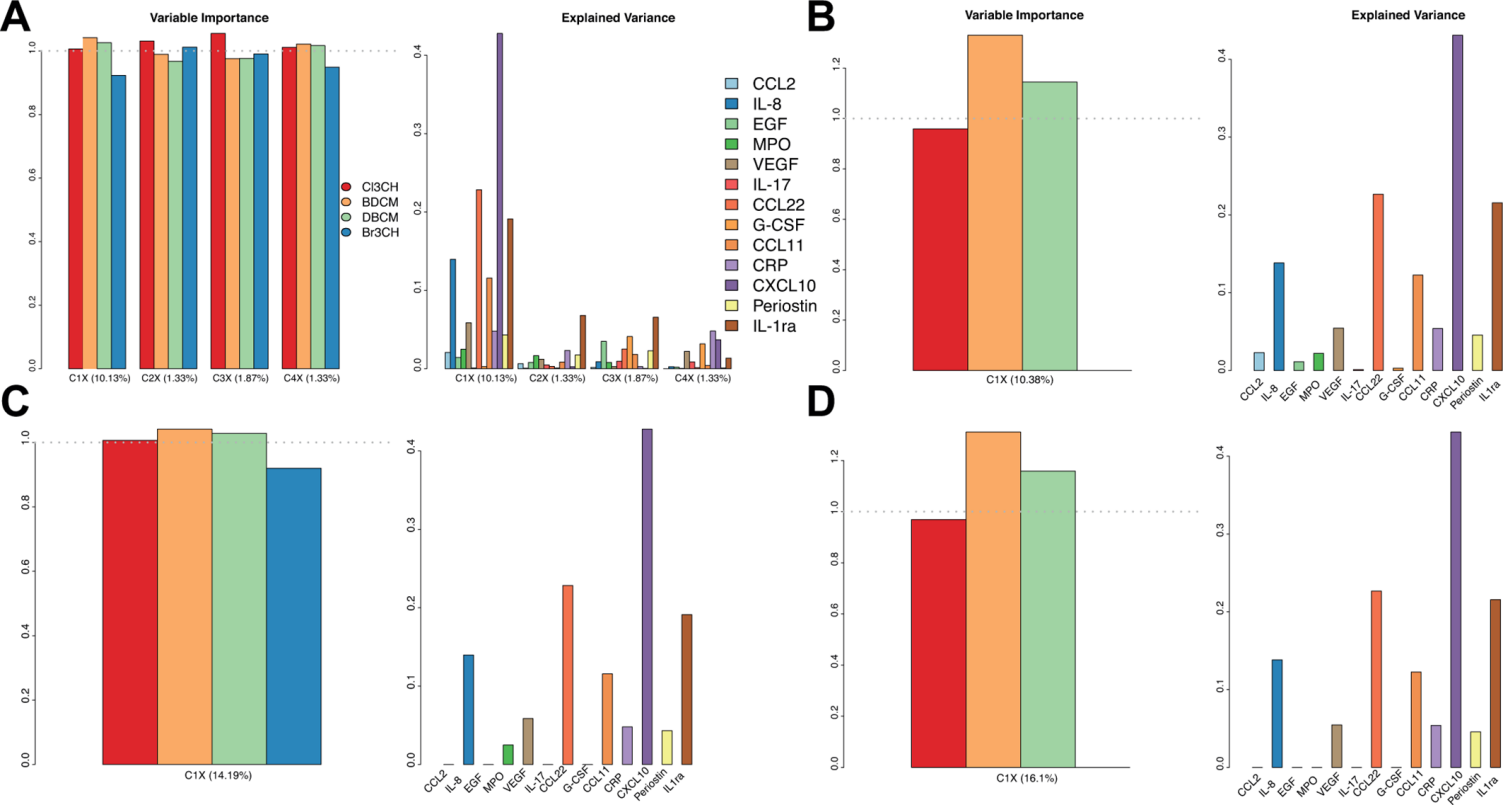
Variable importance in projection plots and proportion of variance explained by protein. Results are presented for PLS model (A), for sparse PLS performing variable selection on exposures (B), on proteins (C), and both on exposures and proteins (D). CCL11, C-C motif chemokine 11, CCL2 motif, chemokine (C-C motif) ligand 2; CCL22, C-C motif chemokine 22; CRP, C reactive protein; CXCL10, C-X-C motif chemokine 10; EGF, epidermal growth factor, G-CSF, granulocyte colony-stimulating factor; IL, interleukin; MPO, myeloperoxidase; PLS, partial least squares; sVEGF, vascular endothelial growth factor.

VIP plots (figure 2A) show that BDC, DCB and to a lesser extent chloroform more importantly contributed to the explanatory performance of C_1X_ (VIP >1) than bromofrom (VIP around 0.9). The second component of X explained <5% of the variance in X which in-turn explained slightly >1% of the variance on Y (table 2), and this was driven by bromoform and chloroform (VIP >1, figure 2A). The proportion of variance explained by each component of X was not homogeneous across proteins and was consistent with the loadings of the Y components (table 2): C_1X_ explained a larger proportion of the variance for CXCL10 (>40%, highest loadings coefficient of C_1Y_: 0.57), CCL22 (>20%, second largest loadings coefficient: 0.42), IL-1ra (around 20%, third highest loadings coefficient in absolute value: −0.38) and IL-8 (around 13%, loadings coefficient: 0.33). All other components explained <10% of each of the original protein levels.

### Variable selection on either X or Y

In order to select the most influential exposures, and the most affected exposures we ran sparse PLS models, with penalisation of the loadings coefficients applied to exposures, and protein respectively. When performing variable selection on exposures, the calibrated sparse PLS model included a single component C_1X_′, in which bromoform was not selected (table 2). C_1X_′ explained 94% of the variance in X and 10.4% of the variance in Y. VIP plot (figure 2B) showed that mainly BDC and DCB contribute to the explanatory performances of C_1X_′ and that this component explains a similar proportion of variance of Y than C_1X_.

The calibrated sPLS models on proteins also included a single component, and in that component four proteins were excluded. These corresponded to those with the least explained variance by exposures in the non-penalised models and models penalised on X (see figure 2A and B, respectively): CCL2, EGF, IL-17 and G-CSF. The first component of X, C_1X_″, was very similar to that of the non-penalised models (table 2), and explained 94.9% of the variance in X, and >14% of the variance of the shrunk set of proteins. As expected, the VIP plot for sPLS models on Y, C_1X_″ (figure 2C), were similar to that of the PLS model C_1X_ (figure 2A), and the proportion of variance explained for each of the selected proteins were not affected.

### Variable selection on both X and Y

In the final model, variable selection is performed on both X and Y, one component is retained for exposures and proteins: C_1X_‴, and C_1Y_‴. As in the sparse model on X, C_1X_‴ does not include bromoform. C_1Y_ ‴ includes eight proteins (vs nine for C_1Y_″) and the five proteins with null loadings were CCL2, EGF, IL-17, G-CSF (as in the sparse on Y model) as well as MPO. C_1X_‴ explains 94% of the variance of X and 16.10% of the variance of the shrunk set of proteins.

In that model, as before, BDCM and DBMC mainly contribute to the explanatory performances of C_1X_‴, and C_1X_‴ explains the variance of eight proteins, mainly: CXCL10 (>40%), CCL22 (>20%), IL1-ra (~20%), IL-8 and CCL11 (~15%) (figure 2D).

Altogether, these sPLS models show that the exclusion of bromoform in the exposure matrix and of CCL2, EGF, IL-17, G-CSF, and in a lesser extend MPO, in the protein matrix, does not affect the performances of the model, which suggests that these excluded variables do not contribute to the exposure-protein relationship, and therefore not to the inflammatory response to DBP.

**Figure 3:**
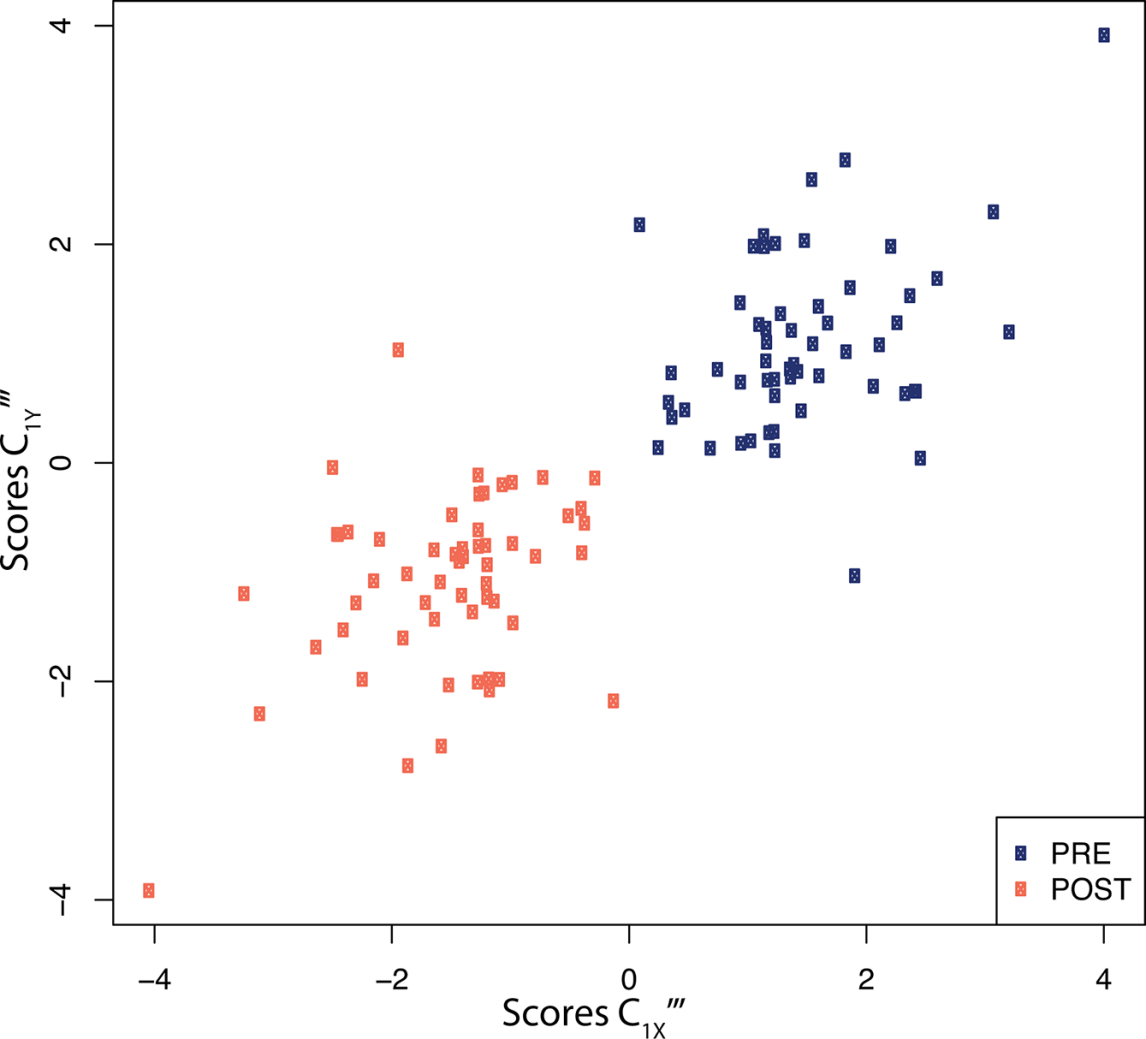
X-Y score plot representing the PLS scores for the first exposure PLS component (C_1X_‴x-axis) as a function of the scores of the first PLS component for proteins (C_1Y_‴, y-axis). Scores are presented for all (n=60) participants before (blue), and after (orange) the swimming session. Results are presented for the sparse PLS models performing variable selection of both exposures and proteins. PLS, partial least squares.

Projection of the dataset using the first PLS components of both exposures and proteins (figure 3) clearly shows that all post-swimming observations exhibit negative PLS scores along the exposure axis. Due to the negative loadings of C_1X_‴ (table 2) this is indicative of increased exposure levels after the swimming experiment. Similarly, all except one of the post-swimming observations are allocated negative PLS scores along the first protein PLS component. This is suggestive of post-swimming decreased levels of IL-8, VEGF, CCL22, CCL11, CRP, and CXCL10 (all have positive loadings coefficients, see table 2), and increased levels of IL-1ra and, to a lesser extent, of Periostin (negative loadings coefficients, table 2).

**Figure 4:**
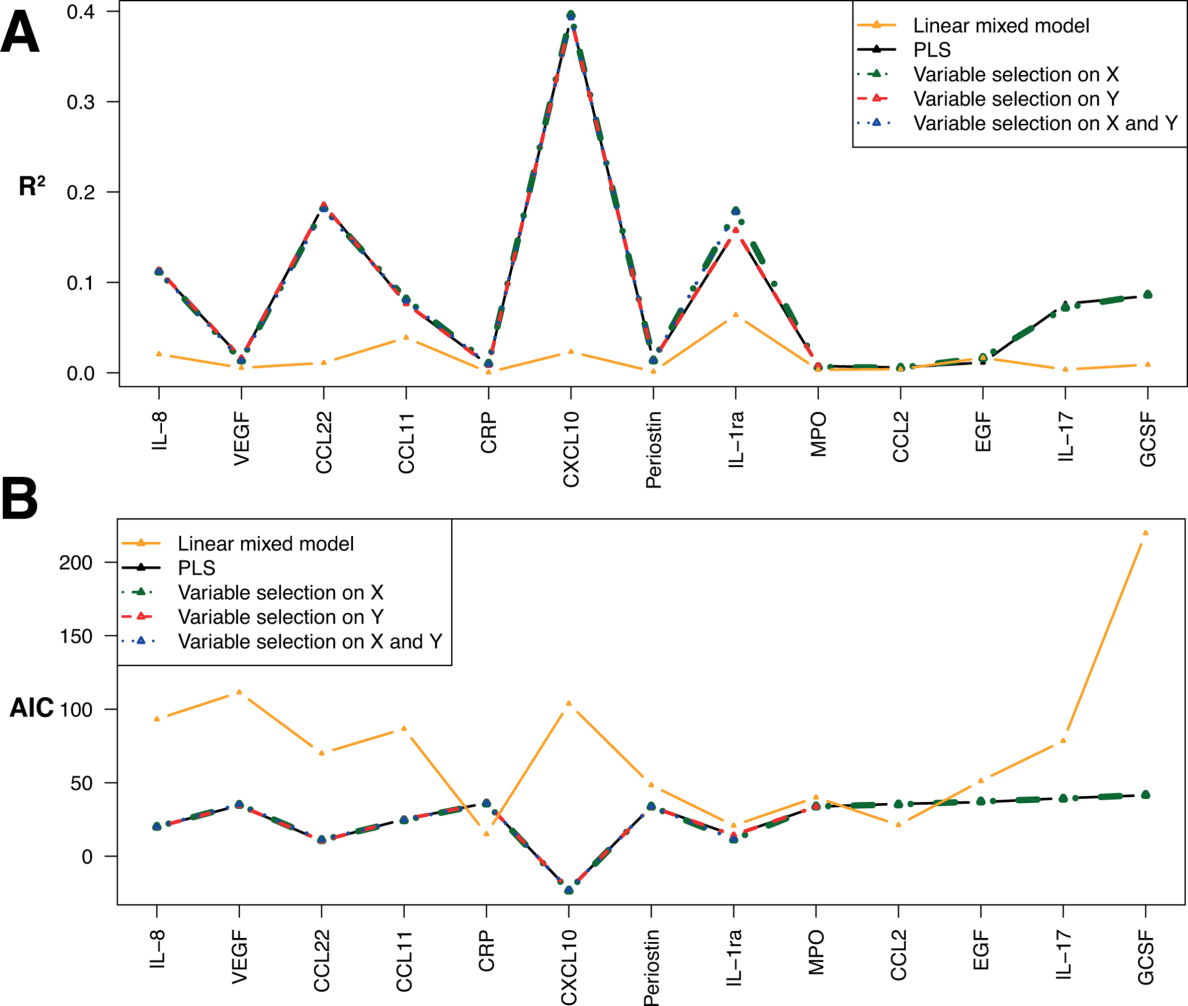
Per-protein coefficient of determination (R^2^) (A) and Akaike information criterion (B) for the four PLS models investigated: non-penalised, with variable selection on X, on Y and on both X and Y. Results are also represented for a linear mixed model using the participant ID as random effect, and the set of four exposure are fixed effects, in relation to each protein separately. CCL11, C-C motif chemokine 11, CCL2 motif, chemokine (C-C motif) ligand 2; CCL22, C-C motif chemokine 22; CRP, C reactive protein; CXCL10, C-X-C motif chemokine 10; EGF, epidermal growth factor, G-CSF, granulocyte colony-stimulating factor; IL, interleukin; MPO, myeloperoxidase; PLS, partial least squares; VEGF, vascular endothelial growth factor.

In figure 4, we compare the per-protein coefficient of determination (R^2^, figure 4A) and the Akaike information criterion (AIC, figure 4B), calculated over the entire study population, for the four PLS models we investigated and for a series of linear mixed models we ran, setting the individual ID set as random intercept to regress all four exposures against each of the protein levels separately. From this plots, it appears that jointly modelling the effect of exposures on all proteins (as done in PLS models) improves the quality of the model fit and yields overall higher R^2^ values and lower AIC for any of the PLS models compared with those from linear mixed models. Additionally, very small differences in both R^2^ and AIC were observed across the four versions of the PLS approaches we used, suggesting that variable exclusion did not affect model performances and that the penalised models efficiently discarded irrelevant exposures, and/or unrelated exposures. For some proteins, in particular CXCL10, IL8, VEGF, CCL22 and CCL11, all PLS models yield better performances than linear mixed models. These proteins are the ones with the largest proportion of variance explained by exposures in the PLS models. Conversely, for some proteins (eg, MPO, CCL2 and EGF), PLS and linear mixed models similarly misperform, and these proteins correspond to proteins whose changes in concentration cannot be explained by exposures: these proteins were not selected in sPLS models.

## Discussion

A common problem in environmental epidemiology is to identify the complex set of exposures affecting biological processes and ultimately contributing to health. Most environmental exposures operate in the form of complex mixtures, including air, food and water.

Unlike recent alternative explicitly modelling the variance among exposures,^34 35^ we used PLS models to identify subsets of exposures and proteins mostly covarying. In the present study, we chose not to explicitly model the exposure mix including its possible interaction in relation to molecular profiles, but considered instead that the set of measured exposures was a better proxy for the exposure mix than each exposure taken separately.

We propose to use an established extension of the PLS model to identify, within the entire set of measured exposures, those that are relevant to the entire set of biomarkers and, using penalisation, to quantify to which extent these exposures contribute to the overall explanatory performance of the model.

While PLS models can natively accommodate high-dimensional data,^36–38^ we chose to use data of limited dimensionality in our illustration to improve visualisation and interpretation of the main output of the methods. Our data featured two measurements per individual and we handled this design by adopting an establishedmultilevel approach decomposing the total variance into ’between' and ’within' individual variations. The former captures differences across individuals while the latter measures variation induced by the experiment (here, swimming) and is therefore adjusted on potential sources of heterogeneity across participants.

We observed modest changes in the proportion of variance explained across the PLS models investigated, and overall a small proportion of the variance of the proteins explained by the exposures. Our analyses identified a subset of eight proteins that were more affected by swimming-induced exposures and suggested a weaker association with bromoform in comparison to other di-bromo derivatives and chloroform. Our results show that variable selection applied to PLS models in presence of correlated predictors efficiently was able to discard (1/4) exposures not contributing (other than through their correlation with the other exposures) to the explanation of the variance of the protein levels, and (5/13) proteins whose variation was not related to the exposures, and hence improves results interpretability. Further improvements in results interpretability could be achieved by adopting orthogonal PLS approaches^19 39^ where the orthogonal (ie, non-predictive) information from the exposures is removed and does not contribute to the construction of the components.

Results from the sparse PLS models selecting both exposures and proteins suggest a swimming-induced immunotoxicity through decreased level of IL-8, VEGF, CCL22, CCL11, CRP and CXCL10, and increased levels of IL-1ra, which antagonises the proinflammatory IL-1. These results are consistent with previous studies showing an anti-inflammatory response in relation to exposures to DBP and/or physical activity.^27–29^ Our results also suggest that these inflammatory changes are concurrent to the acute exposure to DBP.

However, we observe a very strong contrast in exposure levels before and after swimming, which induces a strong correlation between exposures and factors relating to the experiment. Consequently it is not possible to statistically disentangle the effect of exposures from that of physical activity (or of any other factor related to the swimming experiment),^25 28^ and in the absence of independent evidence relating the identified proteins to DBP, these conclusions should be carefully interpreted.

Finally, our results show that adopting a multivariate approach accommodating multivariate predictors and responses (ie, accounting for the variance-covariance across proteins) yields improvement in the model fit (R^2^ and AIC) over models looking at each response separately.

While our example focuses on the immune response to exposure to DBP, this approach could directly be extended to other exposures (in particular air pollutants), and due to the computational scalability of PLS models, such extensions could accommodate other omics data of higher dimensionality.

As such, variants of the PLS approach as used here may complement correlation-based approaches such as the exposome globe^40^ to identify ‘exposome haplotypes’ as well as related phenotypes, which could subsequently be interrogated for (potentially complex) interactions. Once limited to a promising set of exposures, interactions of the mixture components could be explicitly modelled using PLS approaches by including interaction terms in the predictor matrix and define a group structure compiling main effects and interaction terms in the same group, and adopt a sparse group PLS approach.^41^ toThis would identify the relevant sets of exposures (ie, groups) and within each group, the most relevant variables (main and/or interaction terms).

**What is already known on this subject**

- The exploration of the role of exposures on human health relies on the modelling of the effect of exposure mixtures. Several approaches have been proposed to study exposure mixtures, and these rely on strong assumption regarding the identity of the involved exposures and on the form of their interactions. In an exposome context, relevant exposures are not all measured and sometimes unknown, hence making the formulation and justification of such hypotheses impossible.

**What this study adds**

- In order to capture the complexity of the exposure effects and of their biological response, we propose to use PLS approaches combined with variable selection to identify influential subsets of exposures to identify a subset of molecular measurements preferentially affected by these exposures. Our illustrative application investigated the effect of exposure to disinfection by products (n=4) on inflammation, as measured by 13 proteins levels and can naturally be scaled up to high throughput data. It may therefore prove useful in exposome research to explore the complex and possibly pleiotropic effects of exposures on biological makers.
